# Differential value of intima thickness in ischaemic stroke due to large‐artery atherosclerosis and small‐vessel occlusion

**DOI:** 10.1111/jcmm.16884

**Published:** 2021-08-29

**Authors:** Suqin Jin, Cheng Zhang, Yun Zhang, Guoyong Jia, Mei Zhang, Mingjun Xu

**Affiliations:** ^1^ Department of Neurology The Second Hospital of Shandong University Jinan China; ^2^ The Key Laboratory of Cardiovascular Remodelling and Function Research Department of Cardiology Chinese Ministry of Education Chinese National Health Commission and Chinese Academy of Medical Sciences The State and Shandong Province Joint Key Laboratory of Translational Cardiovascular Medicine Qilu Hospital of Shandong University Jinan China; ^3^ Department of Neurology Qilu Hospital of Shandong University Jinan China

**Keywords:** carotid artery, intima thickness, large‐artery atherosclerosis, radial artery, small‐vessel occlusion

## Abstract

No study has examined the differential value of arterial intima thickness in the subtypes of acute ischaemic stroke. This study aimed to assess whether intima thickness of carotid artery (CIT), radial artery (RIT) and dorsalis pedis artery (PIT) have an independent and additive value in differentiating ischaemic stroke subtypes due to large‐artery atherosclerosis (LAA) or small‐vessel occlusion (SVO). One hundred and sixty‐one patients with LAA and 79 patients with SVO were recruited. CIT, RIT and PIT were measured with a 24‐MHz ultrasound transducer. Binary logistic regression analysis was used to evaluate the differential values of the different parameters in the two subtypes. ROC curve analyses were plotted to compare the differential performance of different parameters and the combination model. Both RIT and PIT were substantially thicker in LAA than in SVO stroke patients. RIT and carotid intima‐media thickness had similar performances in differentiating stroke subtypes. Introduction of RIT to traditional atherosclerotic associated risk factors had a marginal satisfactory differential performance for LAA and SVO stroke patients (AUC 0.775). RIT is a promising parameter for LAA and SVO subgroup classification. The combination of RIT and traditional risk factors might be a promising tool for differentiating ischaemic stroke subgroups.

## 1 INTRODUCTION

Cerebrovascular disease is known to be responsible for significant morbidity and mortality worldwide. According to the 2018 World Health Organization Global Health Observatory data, ischaemic heart disease and stroke were the two leading causes of death globally, accounting for a combined 15.2 million deaths in 2016 (https://www.who.int/gho). The aetiologies of ischaemic stroke are heterogeneous. According to an aetiology‐based classification criteria (Trial of Org 10172 in Acute Stroke Treatment, TOAST), stroke subtypes should be classified as large‐artery atherosclerosis (LAA), small‐vessel occlusion (SVO), cardioembolism, stroke of other determined aetiology and stroke of undetermined aetiology,^1^ with the first two being predominant in China.^2^, ^3^

The pathologies in the different stroke subtypes are heterogeneous. Pathological changes in LAA include atherosclerosis,^4^ which occurs in the intima layer of large arteries, including intracranial arteries, as well as extracranial arteries, such as the carotid artery, radial artery and dorsalis pedis artery. In contrast, the pathological changes in SVO include arteriolosclerosis and cerebral amyloid angiopathy. Arteriolosclerosis is mainly characterized by loss of smooth muscle cells from the tunica media, deposition of fibro‐hyaline material in the vessel wall, and thickening of the vessel wall. Meanwhile, cerebral amyloid angiopathy is featured by the progressive deposit of congophilic, βA4 immunoreactive amyloid protein in the walls of small‐medium sized arteries, as well as arterioles.^5^

The differentiation of ischaemic stroke into LAA and SVO subtypes is of great clinical importance in management strategies as well as in prognostication. To date, there are several modalities for differentiation of ischaemic stroke subgroups, including magnetic resonance angiography (MRA), computed tomography angiography (CTA) and transcranial doppler (TCD). MRA and CTA can be used to reveal the angiographical stenosis of cerebral arteries directly; however, both techniques are costly, and allergy to contrast should not be underestimated. TCD can reveal angiographical stenosis indirectly by detecting accelerated blood flow dynamics; however, its precision is limited. In addition, all three techniques are macroscopic in nature, failing to reveal the pathological changes that occur in the arterial intima or media layer microscopically, especially at an early stage of atherosclerosis. There have also been studies involving differentiation of LAA and SVO subtypes with carotid intima‐media thickness (CIMT)^6^; however, CIMT measurement involves combined measurement of both the intima layer and media layer, and its ability to reveal atherosclerosis is limited. Hence, CIMT measurement is no longer recommended to perform in asymptomatic subjects according to the recently published European Society of Cardiology (ESC) guidelines.^7^

Recently, we measured the intima and media thickness of carotid and radial arteries in a group of LAA patients and healthy controls by applying a 24‐MHz high‐resolution ultrasound and found that intima layers were thicker in LAA patients. Such a method could be used to anatomically predict atherosclerosis in the intima layer.^8^ In this study, we aimed to differentiate LAA from SVO by applying the same 24‐MHz frequency high‐resolution ultrasound with the hypothesis that the intima layer is thicker in the LAA subtype than in the SVO subtype, and that it can be used to differentiate the two subtypes of ischaemic stroke.

## 2 MATERIALS AND METHODS

### 2.1 Study population

Three hundred and thirty‐seven consecutive patients in neurology departments of the Second Hospital of Shandong University and Shandong University Qilu Hospital from November 2018 to January 2019 and from April 2019 to June 2019 with a diagnosis of acute ischaemic stroke were initially screened for this study, comprising of 181 LAA patients (53.7%), 92 SVO patients (27.3%), 19 CE (cardioembolism) patients (5.6%), 8 OC (stroke with other determined cause) patients (2.4%) and 37 UND (stroke of undetermined cause) patients (11.0%). Ischaemic stroke was diagnosed according to the presence of a focal neurological deficit lasting for over 24 hours, which was verified by diffusion‐weighted images of magnetic resonance imaging.^9^ Imaging of the carotid and intracranial arteries was accomplished by CTA, MRA or digital subtraction angiography (DSA). Stroke subtypes in this study were classified according to the TOAST classification criteria, which was verified by two experienced neurologists. Patients with multiple vascular lesions were categorized into the LAA stroke group if any one of the arteries manifested as large‐artery atherosclerosis. Patients with a diagnosis of LAA or SVO underwent ultrasound examination. The major exclusion criteria included as follows: ①cerebral haemorrhage, ②atrial fibrillation, ③cardioembolism, ④Takayasu arteritis, ⑤carotid endarterectomy, ⑥uncontrolled severe hypertension (defined as systolic blood pressure>180 mmHg or diastolic blood pressure>120 mmHg) or uncontrolled severe diabetes mellitus (DM), which is defined as DM with acute complications (including diabetic ketoacidosis and hyperosmolar hyperglycaemic non‐ketotic coma), or acute hyperglycaemia state without acute complications (random blood glucose >16.7mmol/L; blood ketone 1.0–3.0mmol/L; or serum osmolality <320 mmol/L), ⑦familial hypercholesterolemia, ⑧valvular disease, ⑨systemic disease, ⑩liver or renal dysfunction, and a poor quality of ultrasound imaging for carotid, radial or dorsal pedal arteries. For LAA patients and SVO patients, 10 and 7 patients were dropped, respectively, because of poor quality of carotid ultrasound imaging, whilst 10 and 6 patients were dropped, respectively, because of exclusion criteria. Two hundred and forty patients with a diagnosis of LAA and SVO were finally enrolled in this study, including 161 LAA and 79 SVO, respectively. LAA patients were further subcategorized into intracranial (ICAS), extracranial (ECAS) and combined (including both intracranial and extracranial, ICAS &ECAS) subgroups according to the location of vascular stenosis. Presence of ICAS was defined as ≥50% stenosis or occlusion in any of the major intracranial arteries in MRA/CTA/DSA, including bilateral intracranial internal carotid arteries, middle cerebral arteries (M1 and M2), anterior cerebral arteries (A1 and A2), posterior cerebral arteries (P1 and P2), vertebral arteries (V4) and basilar artery (BA). Presence of ECAS was defined as ≥50% stenosis or occlusion in bilateral extracranial internal carotid arteries, common carotid arteries and extracranial vertebral arteries.

The study protocol conforms to the ethical guidelines of the 1975 Declaration of Helsinki and was approved by the Ethics Committees of the Second Hospital of Shandong University and Shandong University Qilu Hospital. Informed consent was obtained from all participants.

### 2.2 Demographic features

Data on age, sex, body mass index (BMI), history of smoking, hypertension and DM, use of statins, systolic blood pressure (SBP) and diastolic blood pressure (DBP) were recorded on enrolment. Levels of fasting blood glucose, total cholesterol (TC), serum triglycerides (TG), high‐density lipoprotein cholesterol (HDL‐C), low‐density lipoprotein cholesterol (LDL‐C), small dense low‐density lipoprotein (sdLDL), apolipoprotein A‐1 (ApoA‐1), apolipoprotein B (ApoB), ratio of ApoB/ApoA‐1, lipoprotein (a) (Lp[a]) and non‐esterified fatty acid (NEFA) were also collected.

### 2.3 Arterial ultrasonography and image analysis

The imaging modalities have been described previously.^8^ Briefly, a 24‐MHz frequency high‐resolution linear transducer (PLI‐2004BX) connected to an ultrasound system (Aplio i900, Canon‐Toshiba Ultrasound, Tochigi‐ken, Japan) was used to scan carotid arteries, radial arteries and dorsalis pedis arteries for both left and right sides sequentially. Cine‐loops of at least three consecutive beats were digitally stored with an electrocardiogram simultaneously recorded. The intima and media thickness of the arteries in the far wall were analysed offline with software embedded in the ultrasound machine at the peak of the R wave. Intima‐media thickness was the sum of intima thickness and media thickness. Measurements were performed for carotid intima thickness (CIT), carotid media thickness (CMT), carotid intima‐media thickness (CIMT), radial intima thickness (RIT), radial media thickness (RMT), radial intima‐media thickness (RIMT), podalic intima thickness (PIT), podalic media thickness (PMT) and podalic intima‐media thickness (PIMT). All measurements were performed three times and the mean values were obtained. All offline measurements were performed by a single experienced ultrasonographer blinded to the clinical information.

### 2.4 Statistical analysis

Statistical analysis was performed using SPSS 23.0 (SPSS Inc., Chicago, IL, USA). Continuous data were presented as mean ±SD. Categorical data were presented as numbers (percentages). Kolmogorov‐Smirnov analysis was used to test for normality. An independent t test was performed to compare normally distributed continuous data between groups. Whilst Mann‐Whitney U test was used to compare non‐normally distributed continuous data. The chi‐square test was used to compare categorical data between groups. Binary logistic regression analysis was used to select potential differential parameters for LAA and SVO subtypes. Both forward: LR selection (probability of entry being <0.10) and backward: LR selection (probability of entry and removal being 0.05 and 0.10, respectively) procedures were sequentially performed to select potential differential variables for ischaemic stroke subtypes. Variables significantly contributed to the regression model in both forward: LR selection and backward: LR selection procedures were kept in the combination models and receiver operating characteristic (ROC) curve analysis was performed. A two‐tailed *p*‐value <0.05 was considered statistically significant.

## 3 RESULTS

### 3.1 Demographic features

The demographic features of the LAA stroke group and SVO stroke group are listed in Table 1. There were no significant differences between LAA and SVO subgroups in terms of age, sex, SBP, DBP, BMI, the prevalence of coronary artery disease (CAD), hypertension and serum levels of fasting glucose, TC, TG, HDL‐C, LDL‐C and sdLDL. In patients with LAA stroke, the serum levels of NEFA, ApoA‐1, ApoB and Lp(a) were higher than those in patients with SVO stroke. The prevalence of DM and smoking was also higher in the LAA group than in the SVO group (Table 1).

**TABLE 1:** Demographic, biochemical and ultrasonic data in LAA and SVO groups

	LAA group (n=161)	SVO group (n=79)	*p* Value
Age (year)	62.11 ± 9.66	63.00 ± 11.64	0.533
Male (n, %)	98 (60.9%)	51 (64.6%)	0.399
BMI (kg/m^2^)	25.61 ± 3.83	25.57 ± 3.57	0.948
Smoking (n, %)	56 (35.2%)	39 (49.9%)	**0.049**
DM (n, %)	67 (42.1%)	20 (25.3%)	**0.015**
CAD (n, %)	39 (25.2%)	14 (17.7%)	0.248
Hypertension (n, %)	121 (76.1%)	52 (65.8%)	0.122
SBP (mmHg)	153.33 ± 19.99	152.10 ± 21.39	0.664
DBP (mmHg)	85.62 ± 14.64	84.82 ± 14.59	0.691
Statins (n, %)	36 (22.5%)	10 (12.8%)	0.083
Antihypertensives (n, %)	72 (45.3%)	26 (32.9%)	0.071
TC (mmol/L)	4.28 ± 1.18	4.14 ± 0.92	0.351
TG (mmol/L)	1.54 ± 1.36	1.52 ± 0.99	0.925
HDL‐C (mmol/l)	1.08 ± 0.32	1.07 ± 0.30	0.774
LDL‐C (mmol/L)	2.64 ± 0.96	2.47 ± 0.80	0.193
sdLDL (mmol/L)	0.78 ± 0.39	0.84 ± 0.42	0.345
NEFA (umol/dL)	28.91 ± 40.88	15.13 ± 29.80	**0.010**
APOA‐I (g/L)	1.29 ± 0.25	1.21 ± 0.18	**0.024**
APOB (g/L)	1.02 ± 0.31	0.90 ± 0.24	**0.010**
APOB/APOA‐I	0.82 ± 0.29	0.76 ± 0.22	0.161
LP(a) (nmol/L)	57.24 ± 66.69	32.10 ± 38.41	**0.003**
GLU (mmol/L)	6.47 ± 2.50	6.12 ± 2.49	0.310
CIT (×10^−2^mm)	39.61 ± 12.16	39.39 ± 12.18	0.895
CMT (×10^−2^mm)	51.83 ± 15.28	45.77 ± 13.45	**0.003**
CIMT (×10^−2^mm)	91.44 ± 22.89	85.16 ± 19.38	**0.037**
RIT (×10^−2^mm)	15.24 ± 4.14	12.94 ± 2.95	**0.003**
RMT (×10^−2^mm)	18.71 ± 5.68	17.23 ± 4.08	**0.022**
RIMT (×10^−2^mm)	33.95 ± 8.97	30.16 ± 6.17	**<0**.**001**
PIT (×10^−2^mm)	13.81 ± 4.23	12.05 ± 2.88	**<0**.**001**
PMT (×10^−2^mm)	18.95 ± 5.87	16.59 ± 4.98	**0.002**
PIMT (×10^−2^mm)	32.76 ± 9.07	28.64 ± 6.64	**<0**.**001**

Note

Data were expressed as mean ±SD or n (%). The highlighted bold value were used to clearly show parameters with a *p* value <0.05.

Abbreviations: APOA‐I, Apolipoprotein A‐I; APOB, Apolipoprotein B; BMI, body mass index; CAD, coronary artery disease; CIMT, carotid intima‐media thickness; CIT, carotid intima thickness; CMT, carotid media thickness; DBP, diastolic blood pressure; DM, diabetes mellitus; GLU, fasting glucose; HDL‐C, high‐density lipoprotein cholesterol; LDL‐C, low‐density lipoprotein cholesterol; LP(a), lipoprotein (a); NEFA, non‐esterified fatty acid; PIMT, podalic intima‐media thickness; PIT, podalic intima thickness; PMT, podalic media thickness; RIMT, radial intima‐media thickness; RIT, radial intima thickness; RMT, radial media thickness; SBP, systolic blood pressure; sdLDL, small dense Low‐Density Lipoprotein; TC, total cholesterol; TG, triglycerides.

### 3.2 Ultrasonic measurements in carotid, radial and dorsalis pedis arteries

**FIGURE 1:**
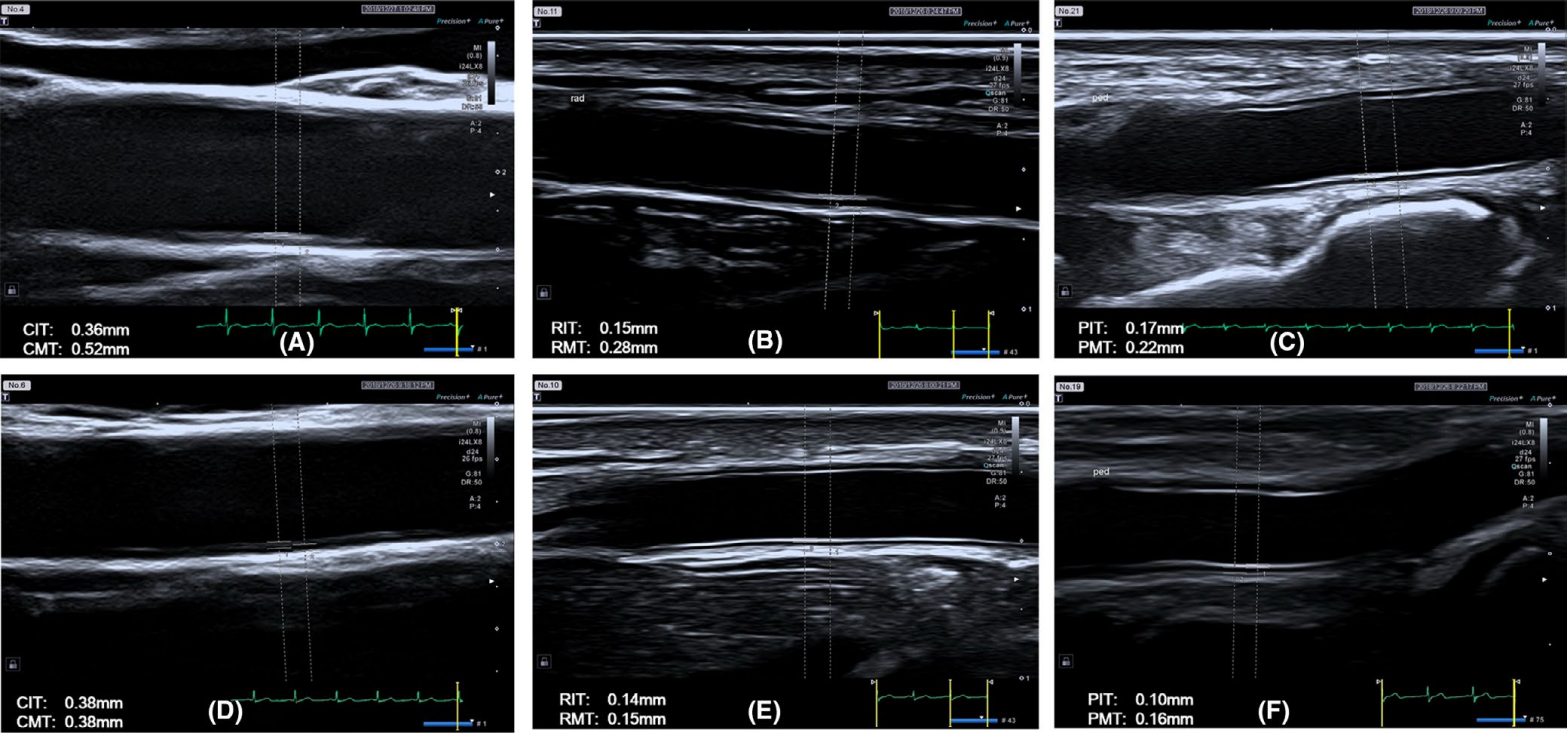
Measurement of the intima thickness and media thickness of the carotid, radial and podalic arteries in a LAA stroke patient and a SVO stroke patient. (A) measurement of the carotid intima thickness (CIT) and media thickness (CMT) in a LAA stroke patient; (B) measurement of the radial intima thickness (RIT) and media thickness (RMT) in a LAA stroke patient; (C) measurement of podalic intima thickness (PIT) and media thickness (PMT) in a LAA stroke patient; (D) measurement of CIT and CMT in a SVO stroke patient; (E) measurement of RIT and RMT in a SVO stroke patient; (F) measurement of PIT and PMT in a SVO stroke patient

Ultrasound measurements in patients with LAA and SVO ischaemic stroke subtypes are also listed in Table 1. Measurements of CMT, CIMT, RIT, RMT, RIMT, PIT, PMT and PIMT were substantially higher in the LAA stroke group than in the SVO group (Table 1, Figure 1). For intracranial LAA subgroup, both CIT and RIT tend to be thinner than those of extracranial and combined groups; however, neither of them was statistically significant amongst groups (Table 2).

**TABLE 2:** Differences of ultrasonic data in LAA patients attributable to intracranial, extracranial or combined artery lesions

	Intracranial (n = 98)	Extracranial (n = 24)	Combined (n = 39)
CIT (×10^‐2^mm)	38.04 ± 12.04	41.29 ± 11.76	42.49 ± 12.37
CMT (×10^‐2^mm)	51.72 ± 15.77	48.88 ± 15.10	53.90 ± 14.16
CIMT (×10^‐2^mm)	89.76 ± 23.52	90.17 ± 22.65	96.38 ± 21.25
RIT (×10^‐2^mm)	15.05 ± 4.08	16.38 ± 5.51	15.03 ± 3.25
RMT (×10^‐2^mm)	18.66 ± 6.04	20.21 ± 5.94	17.90 ± 4.36
RIMT (×10^‐2^mm)	33.71 ± 9.38	36.58 ± 10.28	32.92 ± 6.64
PIT (×10^‐2^mm)	13.81 ± 4.24	13.63 ± 3.92	13.95 ± 4.50
PMT (×10^‐2^mm)	18.80 ± 6.22	19.21 ± 6.57	19.18 ± 4.48
PIMT (×10^‐2^mm)	32.60 ± 9.38	32.83 ± 9.64	33.13 ± 8.07

Note

Data were expressed as mean ±SD.

Abbreviations: CIMT, carotid intima‐media thickness; CIT, carotid intima thickness; CMT, carotid media thickness; PIMT, podalic intima‐media thickness; PIT, podalic intima thickness; PMT, podalic media thickness; RIMT, radial intima‐media thickness; RIT, radial intima thickness; RMT, radial media thickness.

### 3.3 Differentiating value of carotid and radial ultrasound parameters for LAA and SVO subtypes

All parameters, which differed significantly between the LAA and SVO subgroups were introduced into binary logistic regression analysis. After validation with both forward: LR selection and backwards: LR selection, only DM, NEFA, ApoA‐1, Lp(a) and RIT remained in the regression model (Table 3).

**TABLE 3:** Binary logistic regression in differentiating LAA from SVO subtypes

Factors	*β*	SE	Wals	*p* Value	OR	95% CI
DM	1.227	0.444	7.625	**0.006**	3.411	1.428–8.151
NEFA (umol/dL)	0.025	0.011	5.355	**0.021**	1.025	1.004–1.047
APOA−1 (g/L)	2.565	1.017	6.359	**0.012**	13.000	1.771–95.434
LP (a) (nmol/L)	0.011	0.004	6.865	**0.009**	1.011	1.003–1.020
RIT (×10^−2^mm)	0.135	0.058	5.406	**0.020**	1.145	1.021–1.283
Constant	−5.678	1.559	13.265	**0.003**		

The highlighted bold value were used to clearly show parameters with a *p* value <0.05.

Abbreviations: APOA‐1, Apolipoprotein A‐1; CI, confidence interval; DM, diabetes mellitus; LP(a), lipoprotein (a); NEFA, non‐esterified fatty acid; OR, odds ratio; RIT, radial intima thickness; SE, standard error.

**FIGURE 2:**
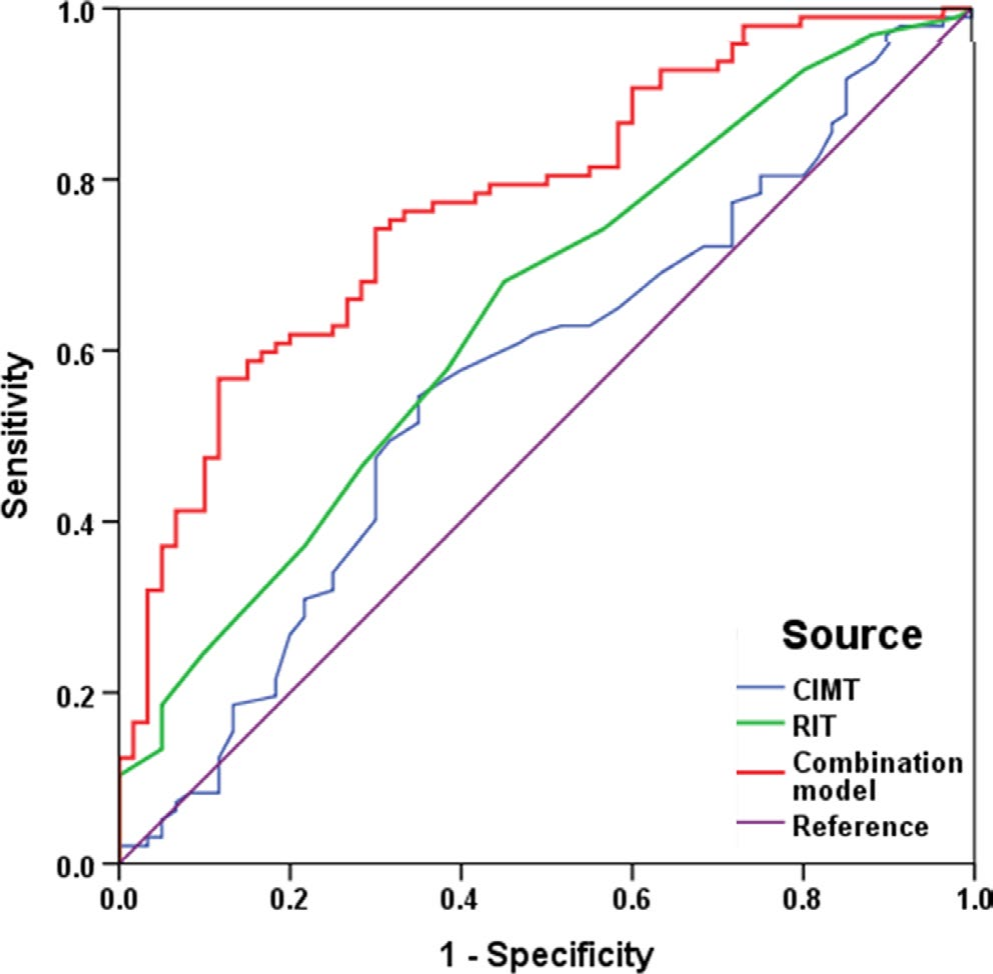
Receiver operating characteristics (ROC) analysis of different variables and combination model for differentiating LAA and SVO stroke subtypes. Combination model contained DM, NEFA, ApoA‐1, Lp(a) and RIT, which yielded the largest area under curve (AUC =0.775)

Based on the above results, we developed a new combination model including DM, NEFA, ApoA‐1, Lp(a) and RIT. Differentiating values of traditional CIMT, RIT and a combination model were plotted with ROC curve analysis. The areas under the ROC curve (AUC) for CIMT, RIT and the combination model were 0.568, 0.646 and 0.775, respectively (Figure 2). Both CIMT and RIT had similar performances in differentiating the two subtypes (AUC: 0.568 vs. 0.646, *p* = 0.23). In contrast, the combination model showed a better differentiating value than traditional CIMT for LAA and SVO stroke subtypes (AUC: 0.568 vs. 0.775, *p* < 0.01), as well as RIT (AUC: 0.646 vs. 0.775, *p* = 0.03). The probability formula used for differentiating LAA stroke from SVO stroke was as follows:

$$p=\frac{e^{‐5.678+1.227X1+0.025X2+2.565X3+0.011X4+0.135X5}}{1+e^{‐5.678+1.227X1+0.025X2+2.565X3+0.011X4+0.135X5}}$$

where X1, X2, X3, X4 and X5 are DM, NEFA, ApoA‐1, Lp(a) and RIT, respectively. Sensitivity and specificity for this new combination model were 74.2% and 70.0%, respectively, when the cut‐off value was 0.566.

## 4 DISCUSSION

To our knowledge, no previous study has evaluated the intima thickness and the media thickness of carotid and peripheral arteries by this novel technique in stroke subtype classification. There were several novel findings in this study. First, the intima and media layers of large and medium arteries were substantially thicker in LAA than in SVO stroke patients. Second, RIT and traditional CIMT have similar performances in differentiating the two subgroups. Third, RIT and traditional risk factors could help differentiate LAA from SVO stroke. Finally, the combination model had the best yet marginally satisfactory differential power for the two subgroups.

Thickened CIMT has long been considered a marker of subclinical atherosclerosis. Previous studies and our recently published data have shown cross‐sectional associations between CIMT and ischaemic stroke,^8^, ^10^, ^11^, ^12^ and the value of CIMT in predicting cerebrovascular diseases.^13^ However, the relationship between CIMT and stroke subtypes remains controversial. Some studies found that CIMT was higher in large‐vessel than in small‐vessel stroke patients,^6^, ^14^, ^15^, ^16^ whilst others found no difference.^17^ This paradox indicated that CIMT alone might not be an ideal parameter to reveal pathological changes of different stroke subgroups and for stroke subtype classification. In addition, thickened intima‐media thickness (IMT) encompasses not only intimal thickening but also medial hypertrophy, which is in response to long‐term hypertension. With the high‐resolution ultrasound transducer, we were able to measure intima thickness and media thickness separately and found that not only CIMT but also CMT, RIT, RMT, PIT and PMT were different between LAA and SVO stroke, indicating that these parameters might serve as potential candidates for stroke subtype classification. Surprisingly, there was no difference in CIT between the two groups. This might be due to several reasons. First, the carotid artery is a large elastic vessel, whilst the intracranial arteries are mainly medium‐sized muscular vessels, and there are differences in anatomical structures. Secondly, both CIT and RIT were reported to be related to age, SBP and HDL‐C^8^; however, there was no difference between the LAA and SVO subgroups regarding all the above‐mentioned parameters in this study. Thirdly, the pathologies in LAA and SVO are complicated, and atherosclerosis might contribute to both subtypes; the carotid artery is more vulnerable to atherosclerosis anatomically in nature and blood flow dynamics, leading to poor differentiating value of carotid parameters for the two groups.

In this study, we found that RIT might help to differentiating between LAA and SVO stroke subtypes with modest differential power (AUC: 0.646). Similar performance of RIT was noticed in our previous study.^8^ In addition, RIT was reported as a predictor of adverse cardiovascular events, including stroke.^18^ This indicates that RIT could be useful in diagnosis and outcome prediction of atherosclerosis‐related diseases. This might be due to the similarity in the anatomical structure of the radial, cerebral and coronary arteries. All three arteries are medium‐sized muscular arteries with similar vulnerabilities to atherosclerosis. Some studies have attempted to identify differential parameters (such as the percentage of brachial flow‐mediated vasodilatation and pulse wave velocity) amongst stroke subtypes by measuring peripheral arteries.^17^, ^19^, ^20^ However, these parameters, reflecting arterial stiffness and endothelial function, were largely functional and likely influenced by various factors.

In this study, we developed a new comprehensive model for the differentiation of LAA and SVO stroke subtypes by combining traditional risk factors with high‐resolution ultrasound parameters. The new combination model showed a marginal satisfactory differential value (AUC: 0.775), which was significantly better than that of traditional CIMT. This new methodology provides a promising tool for stroke subgroup classification.

The findings of this study have important clinical implications. An increased CIT and RIT measured by a 24‐MHz ultrasound probe may be the earliest visible signs of atherosclerosis in humans, well before the stage of thickened IMT and plaque formation. In view of the recent finding that CIMT measurement showed no additive value to the Framingham risk score for cardiovascular event prediction^21^ and the fact that measurement of CIMT is not recommended for asymptomatic subjects in the latest published ESC guidelines,^7^ RIT may replace CIMT in the prediction of long‐term cardiovascular risk. In addition, this novel parameter can be used as an early indicator of atherosclerosis and a predictor of less accessible cerebral vessel disease. As the ultrasonic measurement of CIT and RIT is non‐invasive, this method can be readily used in population studies. Once the prognostic significance of increased CIT and RIT is confirmed in a longitudinal study of a large cohort of patients, these novel measurements may become an early preventive and therapeutic target for atherosclerosis, including ischaemic stroke, and a differential tool for stroke subtype classification.

There was no difference of CIT and RIT amongst intracranial, extracranial and combined subgroups for LAA, indicating both CIT and RIT might be a promising parameter for LAA and SVO subtype differentiation; however, a large sample‐sized study is needed to warrant this conclusion.

There were several limitations to this study. First, the sample size of this cohort was still relatively small, which hindered further analysis regarding the location of infarction or the number of culprit arteries. Secondly, our study is a cross‐sectional study without follow‐up information; therefore, the predictive values of these novel ultrasound parameters are not investigated. Thirdly, other subtypes of ischaemic stroke, such as cardioembolism were not included. These limitations call for a multi‐centred large‐sample sized study with follow‐up information to ascertain the prognostic values of CIT and RIT in ischaemic stroke patients.

In conclusion, RIT is a promising parameter for LAA and SVO subgroup classification. The combination of RIT and traditional risk factors might be a promising tool for differentiating ischaemic subtypes.

## CONFLICT OF INTEREST

The authors confirm that there are no conflicts of interest.

## AUTHOR CONTRIBUTIONS

**Suqin Jin:** Formal analysis (equal); Investigation (equal); Methodology (equal); Writing‐original draft (equal). **Cheng Zhang:** Conceptualization (equal); Writing‐review & editing (equal). **Yun Zhang:** Conceptualization (equal); Writing‐review & editing (equal). **Guoyong Jia:** Investigation (equal); Methodology (equal). **Mei Zhang:** Conceptualization (equal); Writing‐review & editing (equal). **Mingjun Xu:** Conceptualization (equal); Formal analysis (equal); Investigation (equal); Methodology (equal); Supervision (equal); Writing‐original draft (equal).
